# The spontaneous electrical activity of neurons in leech ganglia

**DOI:** 10.1002/phy2.89

**Published:** 2013-09-23

**Authors:** Majid Moshtagh-Khorasani, Evan W Miller, Vincent Torre

**Affiliations:** 1 Neuroscience Area, International School for Advanced Studies (SISSA)via Bonomea, 265, Trieste, 34136, Italy; 2 Department of Pharmacology, University of California at San DiegoLa Jolla, San Diego, 92093, California

**Keywords:** Electrical activity, leech ganglion neurons, voltage-sensitive dye

## Abstract

Using the newly developed voltage-sensitive dye VF2.1.Cl, we monitored simultaneously the spontaneous electrical activity of ∼80 neurons in a leech ganglion, representing around 20% of the entire neuronal population. Neurons imaged on the ventral surface of the ganglion either fired spikes regularly at a rate of 1–5 Hz or fired sparse spikes irregularly. In contrast, neurons imaged on the dorsal surface, fired spikes in bursts involving several neurons. The overall degree of correlated electrical activity among leech neurons was limited in control conditions but increased in the presence of the neuromodulator serotonin. The spontaneous electrical activity in a leech ganglion is segregated in three main groups: neurons comprising Retzius cells, Anterior Pagoda, and Annulus Erector motoneurons firing almost periodically, a group of neurons firing sparsely and randomly, and a group of neurons firing bursts of spikes of varying durations. These three groups interact and influence each other only weakly.

## Introduction

The understanding of the nervous system, from the perspective of systems neuroscience, requires the identification of patterns of electrical activity associated with sensory perceptions leading to specific behavioral reactions (Nicholls et al. 2011). Patterns of electrical activity can be recorded with multiunit electrodes (Takehara-Nishiuchi and McNaughton 2008; Luczak et al. 2013) and/or using imaging tools (Grinvald and Hildesheim 2004; Kerr and Denk 2008; Wallace et al. 2008; Bonifazi et al. 2009) allowing the recording of the electrical activity of some hundreds of neurons. Neuronal networks in the vertebrate brain, however, are composed of millions and often billions of neurons and the present technology allows the monitoring of an extremely limited proportion of these neurons. Simpler nervous systems, such as those of invertebrates, are composed of a significantly smaller number of neurons and therefore it is possible to record the electrical activity of a much larger proportion of neurons forming the network.

The central nervous system of the leech is composed of a chain of 21 ganglia, each of which has ∼400 neurons (Macagno 1980). In each ganglion sensory inputs – primarily mechanical stimulation of the skin and body – are transduced by seven pairs of mechanosensory neurons; three specific for light pressure (touch or T cells), two for strong pressure (pressure or P cells), and two for noxious mechanical stimuli (N cells), (Nicholls and Baylor 1968; Kristan 1982; Lewis and Kristan 1998a,b; Pinato and Torre 2000; Pinato et al. 2000; Arisi et al. 2001). Leech motor reactions are mediated by 21 pairs of excitatory motoneurons and seven pairs of inhibitory motoneurons are present in all ganglia (Mason and Kristan 1982; Norris and Calabrese 1987; Lockery and Kristan 1990a,b). These motoneurons have been extensively investigated using force and length transducers, imaging of muscle contractions and other electrophysiological tools (Stuart 1970; Ort et al. 1974; Kristan 1982; Mason and Kristan 1982; Norris and Calabrese 1987; Zoccolan et al. 2001, 2002; Zoccolan and Torre 2002; Garcia-Perez et al. 2004). In each ganglion, sensory neurons are connected to motoneurons through a network of some hundreds of interneurons receiving inputs also from neighboring ganglia (Kristan et al. 2005).

The spontaneous electrical activity of the leech nervous system has been analyzed using suction pipettes recording the firing of motoneurons innervating the nerves (Mazzoni et al. 2007). These recordings are characterized by irregular bursts of spikes with variable duration and size. In this investigation it was possible to record the spontaneous electrical activity for several hours, but the analysis was restricted to motoneurons, representing only a subpopulation of leech neurons. Therefore, it was not possible to determine whether bursts were part of a more global electrical activity involving the entire leech ganglion or were segregated. Voltage-sensitive dyes have been used in the leech nervous system before but because of their limited temporal resolution only slow changes of the membrane voltage could be detected (Briggman et al. 2005; Briggman and Kristan 2006).

For the present study, we recorded and analyzed patterns of electrical activity using a new generation of voltage-sensitive dyes called VF2.1.Cl. The VF2.1.Cl dye detects voltage changes in neuronal membrane potentials by modulating the photo-induced electron transfer (PeT) from an electron donor through a synthetic molecular wire to a fluorophore (Miller et al. 2011). Voltage-sensitive dyes have been used to study the electrical activity of population of neurons in invertebrates (Cohen et al. 1989; Frost et al. 2007; Stein et al. 2011; Städele et al. 2012), primarily for the detection of synaptic potentials and spikes in large neurons. This dye provides larger responses with a faster kinetics than other voltage-sensitive dyes, allowing the monitoring of slow and fast changes of voltage membrane larger than 3–5 mV. Therefore, a more complete description of the spontaneous activity of leech neurons not restricted to motoneurons (Mazzoni et al. 2007) is obtained. In several experiments, it was possible to monitor the electrical activity of about 80 neurons, that is, ∼20% of the neurons present on each side of a single leech ganglion. The effect of serotonin as a modulator of the spontaneous electrical activity has also been studied. Here, we analyze in detail the cross-correlation structure of the electrical activity by computing the cross-correlation among spikes and graded signals. We show two main properties of the spontaneous electrical activity of the leech nervous system: first, in the absence of sensory stimulation the firing of leech neurons is poorly correlated; second, bursts of electrical activity are segregated and restricted to a small portion of a leech ganglion.

## Methods

The experiments described here were obtained from the isolated single ganglion of the leech *Hirudo medicinalis* ordered from Ricarimpex (Eysines, France). Leeches were maintained at 5°C in tap water dechlorinated by previous aeration in which Instant Ocean salt was diluted. (0.5 g/L; Aquarium Systems, Sarrebourg Cedex, France). Before each experiment leeches were kept at room temperature for 24 h.

### Intact and desheathed ganglia

**Figure 1:**
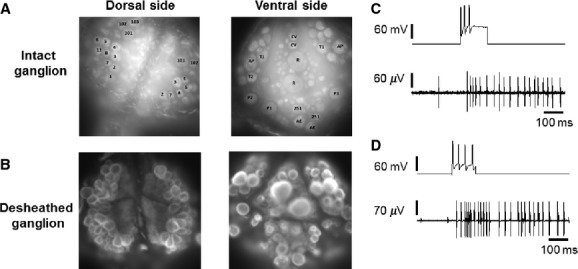
Preparation. (A) images of a dorsal and a ventral surface of an intact leech ganglion, stained with the voltage-sensitive dye VF2.1.Cl. (B) as in A, but after desheathing the ganglion. (C) Upper trace: intracellular recording from a P cell in an intact ganglion, during which the sensory neuron was depolarized by injecting a pulse of current through the microelectrode. The injected current pulse evoked a train of spikes in the sensory neuron, which elicited a train of spikes recorded extracellularly with a suction electrode from a DP nerve (lower trace). (D) as in C but in a desheathed ganglion; a train of spikes in a P cell evoked a train of spikes in the DP nerve.

Before each experiment, animals were anesthetized by chilling with ice for 15–20 min. Leeches were then immersed in 150–200 mL chilled normal ringer solution (in mmol/L: 115 NaCl, 1.8 CaCl_2_, 4KCl, enriched with 10 glucose and buffered with 10 Tris-maleate pH 7.4 with NaOH). Leeches still under anesthesia were pinned with fine needles in their midbody. During the dissection, the temperature was maintained at 6–8°C using a cold chamber. Isolated ganglia (ganglia 8–16) dissected from the nerve cord were transferred and pinned in a Petri dish coated with a silicone elastomer (Sylgard 184; World Precision Instruments, Sarasota, FL), then stained with voltage sensitive dyes (VSDs). Neurons in these stained ganglia were visible and could be properly identified (Fig. 1A), but it was difficult to obtain good optical signals from these ganglia unless we removed the sheath enveloping the ganglion. For desheathing, we used an ultra fine micro knife (from Fine Science Tools, Heidelberg, Germany) to remove the connective-tissue capsule and the layer of enveloping glial cells (Baylor and Nicholls 1969). Desheathed ganglia preserved the original neuronal organization (Fig. 1B).

To verify that the desheathing procedure did not alter the functional connectivity among neurons, we compared the electrical activity, recorded from the nerves, evoked by the firing of 3–4 SPIKEs in P sensory neurons in intact (Fig. 1C) and desheathed ganglia (Fig. 1D); the evoked activity was very similar in intact and desheathed ganglia. Experiments were performed at room temperature (19–22°C).

### Dye loading and imaging with VSD

The voltage-sensitive dye was diluted with normal Ringer solution to the desired concentration (400–500 nmol/L), and 1.5 μL of a 20% (w/v) solution of Pluronic F-127 in DMSO (Miller et al. 2011).

The solution containing the dye was pumped through the desheathed ganglion by a peristaltic pump for 20–30 min. After dye loading, the ganglion was rinsed with the usual saline solution (see above). Before imaging, the silicone layer to which the ganglion was pinned was cut and transferred to a glass bottom petri dish containing 4–5 mL of normal ringer solution and turned upside-down so that the ganglion faced the objective of the inverted microscope. Imaging was obtained using an Electron Multiplier CCD Camera C9100-13 from Hamamatsu Photonics equipped with the Wasabi software (Düsseldorf, Germany). Images were acquired at a sampling rate varying from 94 to 110 frame/s and at a spatial resolution of 128 × 128 pixels. A Nikon Eclipse Ti inverted Microscope with the 20×/0.75 objective was used and the sample was illuminated with a 488 nm light, emitted by a HBO 103 W/2 mercury short arc lamp from Osram (Munich, Germany). The excitation light intensity was attenuated by putting Nikon neutral density filters, ND4 and ND8, after the light source. Emission was collected with a 505 nm long pass filter, after the light passed through a 490 nm dichroic\beamsplitter.

We found convenient to have a series of optical recordings lasting 30 or 60 sec interspersed by 1 or 2 min of darkness, during which the ganglion and the dye could adjust to photobleaching. Therefore, we were able to obtain reliable optical recordings for ∼10 min. There was no significant difference in the firing rate of the Retzius cells, Annulus Erector (AE), and Anterior Pagoda (AP) neurons in control experiments in the absence of the VSD (16 experiments) and in the presence of the VSD (22 experiments), suggesting limited toxicity of the dye during our optical recordings.

### Electrical recordings

We made suction pipettes from borosilicate glass capillaries (World precision instruments, Germany) pulled by a conventional puller (P-97; Sutter Instruments Co., Novato, CA). The electrode tip was cut using a diamond sharpened tip mounted on an appropriate manipulator under a stereoscopic microscope (Olympus SZ40, Shinjuku, Tokyo, Japan). Electrodes with a final internal diameter between 80 and 150 μm, were polished using an incandescent filament under a 20× objective mounted on an upright microscope (Zeiss, Oberkochen, Germany). Suction electrodes filled with normal ringer leech solution were connected to an extracellular recording amplifier (Pinato and Torre 2000; Pinato et al. 2000). Extracellular signals were digitized at 10 kHz by an A/D converter (model digidata-1322, 16 bit converter; Axon, Molecular Devices, Sunnyvale, CA) and data were transferred and stored on a PC computer. Signals were recorded and visualized using, respectively, Clampex v.8.1 and Clampfit v.9.2 software (Molecular Devices). Extracellular electrical recordings were obtained from cleaned nerves of a single ganglion using two suction pipettes sucking the left and right dorsal-posterior (DP) nerves (Pinato et al. 2000; Arisi et al. 2001). We impaled neurons with a sharp intracellular microelectrode (30 MΩ filled with 4 mol/L potassium acetate), as previously described (Pinato et al. 2000; Arisi et al. 2001).

### Processing and analysis of optical recordings

The initial processing of video images was performed using the Wasabi software, provided by Hamamatsu. Image sequences were also processed and analyzed by a software developed in house. For the analysis we used a program written in MATLAB version 7.11. Neurons were imaged and an appropriate region of interest (ROI) around the neuron cell body was selected. The time course of the fluorescence intensity I_f_(*t)* in this ROI was displayed on the computer screen and the extent of its decay – consequence of dye bleaching – was evaluated. To compensate photo bleaching, the decay of I_f_(*t*) was fitted with an exponential function or with polynomial or cubic splines. The compensation based on the exponential function fitted the decay of fluorescence intensity I_f_(*t*) with the single exponential function:


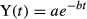
(1)

When the decay of I_f_(*t*) was not well fitted by the single exponential function we used either a third order polynomial:


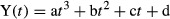
(2)

or cubic splines interpolating I_f_(*t*) at 10 or 20 points. The function Y(*t*) fitting I_f_(*t*) was then added to the original optical signal – to compensate dye bleaching – and the fractional optical signal was taken as (Y(*t*)-I_f_(*t*))/I_f_(0), where I_f_(0) is the fluorescence intensity at the beginning of the recording.

### Computation of raster plots

**Figure 2:**
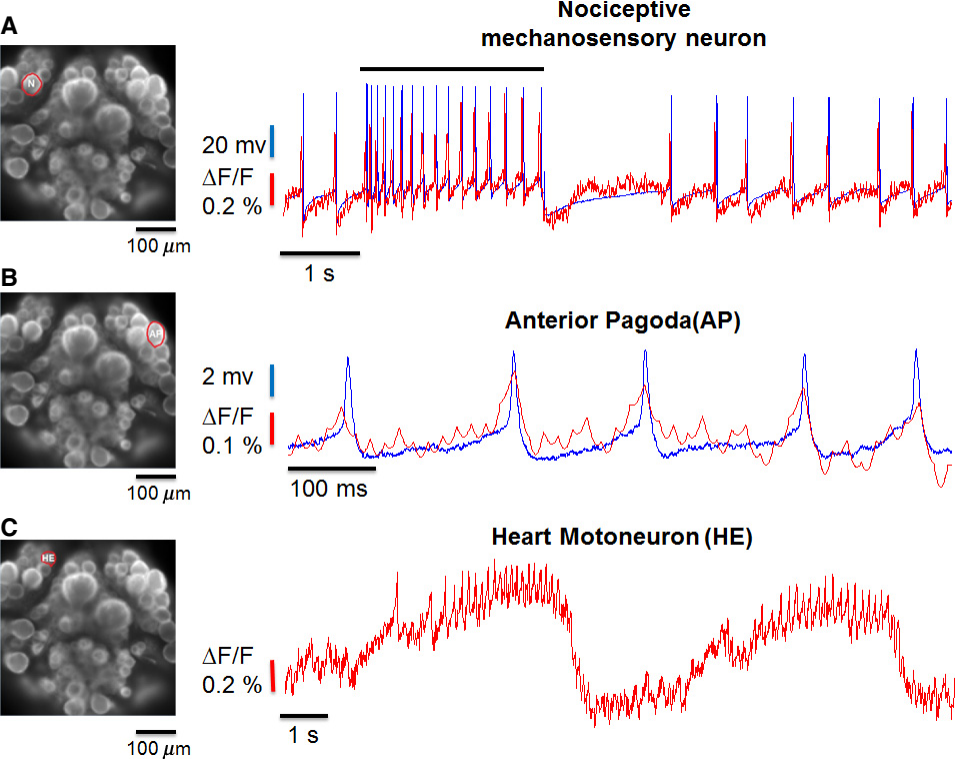
Comparison of optical (red) and electrical (blue) recordings and optical recordings from the heart motoneuron (HE). (A) Simultaneous recordings from the mechanosensory neuron encircled in red in the panel on the left. Images acquired at 100 Hz. Optical and electrical recordings have the same time course, but the peak of the spike of the mechanosensory neuron has a variable amplitude because of the limited sampling rate of image acquisition. Optical recordings follow very precisely the undershoot of about 10 mV following the spike. (B) as in A, but during an electrical recording from the Anterior Pagoda (AP) neuron encircled in the panel on the left. The amplitude of spike of AP neurons, recorded in the soma, is ∼4 mV and is clearly recorded also during the optical recording. (C) Optical recording from the Heart Motoneuron encircled in the panel on the left. The optical recording exhibits an oscillatory behavior with a period of 5–10 sec typical of these neurons (Kristan et al.^2005^) during which the membrane potential changes by about 10 mV.

**Figure 3:**
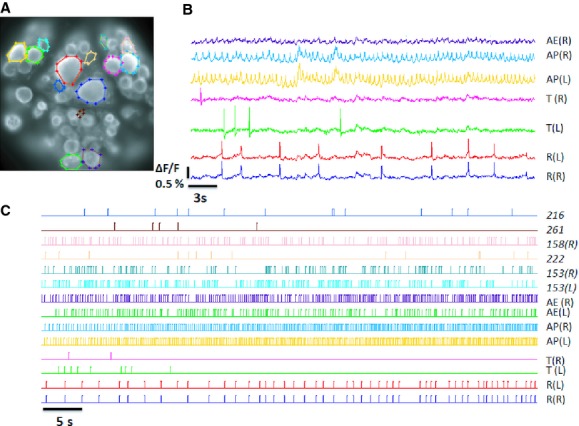
Optical recordings of the spontaneous electrical activity from the ventral surface of a leech ganglion. (A) An image of a ventral surface of the ganglion from which the optical recordings shown in B were obtained. The color of each line encircling a neuron in A corresponds to the optical recordings in B. (B) ΔF/F recordings obtained during spontaneous activity of the ganglion. (C) Raster plots of detected spikes from the obtained ΔF/F recordings. Neurons were identified by their location in the ganglion according to the classical atlas of leech neurons (Muller et al. 1981). The shape of recorded spikes provides an additional check for the identification of neurons, such as Retzius, APs, AEs, and mechanosensory neurons. The identification of neurons indicated in *italics* is tentative. The letters R and L in parenthesis indicate whether the neuron was located on the right or left side of the ganglion.

We detected the spikes in the optical traces in two ways. First, when the spikes were clearly evident, as those from Retzius cells and mechanosensory neurons (Figs. 2 and 3), we manually set a threshold Th and the spikes were identified to occur at the times *t*_i_ corresponding to the crossings of Th. These times were stored as a file. In optical recordings from neurons known to produce spikes with an amplitude of less than 10 mV (APs, AEs, motoneurons, and interneurons) the standard deviation σ_o_ of the optical trace was estimated and upward deflections of the trace larger than 5σ_o_ were identified as spikes. Upward deflections lasting longer than 10 msec, that is, for two successive frames, were not considered to be spikes. Detected spikes were also visually controlled by the experimenter. These times *t*_i_ were finally represented in conventional raster plots.

If the average firing of a neuron over a total recording time of at least 2 min was Xspikes/sec, we defined a burst as the sudden increase of the firing frequency above 5Xspikes/sec (see Fig. 6).

### Comparison of optical and electrical recordings

Several neurons were impaled with a fine intracellular microelectrode, while simultaneously acquiring optical signals. As shown in Figure 2A, the optical trace (red line) superimposed quite well onto the electrical recording (blue line) obtained with an intracellular electrode. When an N mechanosensory neuron was impaled, they generated spikes with an amplitude up to 100 mV that were clearly seen in the optical signal. The signal to noise ratio (S/N) increases with the number of pixels composing the ROI corresponding to the cell body of the neuron: for large Retzius cells the ROI was composed of 100–200 pixels and for the smaller neurons, such as an N cell, the ROI was composed of 80–120 pixels. The amplitude of spikes in Retzius and N cell are 30 and 80 mV, respectively, and are detected optically with a signal to noise of 5 and 6, respectively, and the fractional change of measured fluorescence was ∼1% per 100 mV. Using an acquisition rate of 100 frames per second we detected spikes from APs, AE neurons, which have an amplitude of only 4–6 mV (Fig. 2B). Under these conditions, we could reliably record optical signals also caused by slow changes of the membrane potential and when the heart motoneuron (HE) was imaged (Fig. 2C) we optically recorded slow waves that had a period of about 5 sec, during which small spikes could be seen riding on top of positive deflections. These optical traces had time courses very similar to those obtained with intracellular recordings (Kristan et al. 2005) where the membrane voltage undergoes cyclic oscillations with an amplitude of about 10 mV. These results indicate that the dye VF2.1.Cl is able to detect changes of the membrane potential with an amplitude of 5 mV or less with a S/N larger than 2–3.

### Computation of cross-correlation of occurrence of spikes

The cross-correlation 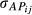 of the firing of neuron *i* and neuron *j* was computed in the following way. The total recording time *T*_*tot*_ was divided into *N* intervals (1, .., *n*,…, *N*) of a duration Δt and if *f*_in_ and *f*_*jn*_ are the number of spikes fired by neuron *i* and neuron *j* in the time interval Δt_*n*_, then


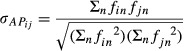
(3)

so that 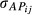 depends on Δ*t* and varies between 0 and 1. The range of explored values of Δ*t* ranged from 10 to 200 msec.

### Computation of cross-correlation of slow signals

It is well known that several leech neurons do not have an excitable cell body and very often spikes are produced only in distal dendrites, therefore it is useful to investigate also the cross-correlation among slow or graded signals, keeping in mind that the cross-correlation among slow/graded signals can be negative, while 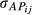 varies only between 0 and 1. The cross-correlation 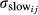 of slow signals obtained from neuron *i* and neuron *j* was computed in the following way. If *s*_in_ is the slow signal from neuron *i* at time *t*_*n*_ its mean value <*s*_*i*_> is computed as 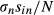 where *N* is the total number of available samples.


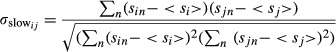
(4)

so that 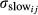 varies between −1 and 1 and the typical interval on which 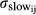 was computed varied from 30 to 180 sec. *s*_in_ was obtained by smoothing, using a time window of 100 msec, to remove spikes from the optical signal.

## Results

The new VSD VF2.1.Cl allows the detection of spikes and also of slow changes of the membrane voltage (see Fig. 2), which cannot be resolved neither by calcium imaging (Grienberger and Konnerth 2012) nor by multielectrode recordings of extracellular voltage signals (Takehara-Nishiuchi and McNaughton 2008; Luczak et al. 2013). The VSD VF2.1.Cl allows satisfactory optical recordings for some minutes, up to a maximum of 10–15 min. As shown in Figure 1, in order to obtain satisfactory optical recordings, it is necessary to desheath the ganglia, a surgical operation which could slightly displace neurons from their original location inside the ganglion. The VSD VF2.1Cl allows the simultaneous recordings of electrical signals in leech ganglia with an unprecedented resolution and accuracy.

In the present investigation, we analyze the spontaneous electrical activity of the leech nervous system, that is, in the absence of sensory stimulation and when the animal is not engaged in a specific behavior, such as swimming or crawling. The characterization of the spontaneous activity is equivalent to the analysis of the background noise and it is a necessary step for a proper understanding of information processing in this nervous system.

### Optical recordings from the ventral surface

We imaged the ventral surface of the ganglion, focusing on as many neurons as possible (Fig. 3A). The two largest neurons in the middle of the ganglion are the Retzius cells, with somewhat smaller mechanosensory neurons on either side (Muller et al. 1981). Optical recordings with the voltage-sensitive VF2.1.Cl obtained from the ventral surface of a leech ganglion (Fig. 3B) detected well-resolved spikes from the soma of Retzius cells, mechanosensory neurons, AP, AE, and Leydig neurons, and also from other interneurons tentatively identified as cells 153, 158, 216, 222, and 261. A typical raster plot of detected spikes is shown in Figure 3C. In all preparations Retzius cells, AP neurons, and AE neurons fired almost continuously at a frequency varying from 0.5 to 7 Hz, as did two other neurons, identified as cells 153 and 158 based upon their location in the ganglion and size (Muller et al. 1981). While neurons 153 and 158 fired almost continuously, irregular spikes were observed in other neurons, tentatively identified as neurons 216, 222, and 261 (Muller et al. 1981).

The spontaneous activity of neurons imaged on the ventral surface of the leech ganglion had a rather stereotyped pattern: several neurons fired spikes almost continuously, such as the 2 Retzius cells, the 2 APs, the 2 AEs, interneurons 153 and 158. The remaining neurons either fired sparse, low-amplitude spikes or were nonspiking neurons. We never observed bursts of spikes in any neurons imaged on the ventral surface of a leech ganglion, with the exception of mechanosensory T cells. This pattern was consistently observed in 22 isolated leech ganglia stained with the VSD VF2.1.Cl.

### Firing and correlation pattern of electrical signals in the ventral surface

**Figure 4:**
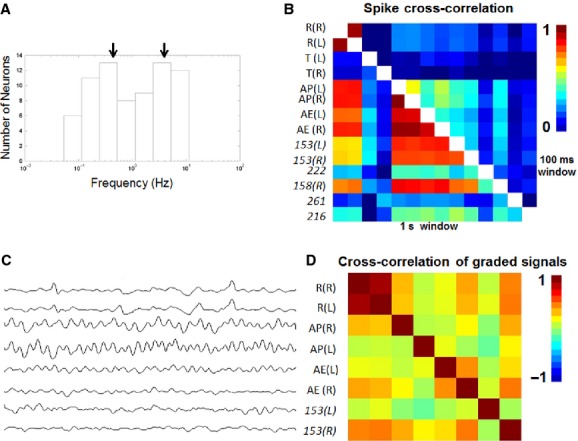
Correlation structure of electrical activity of neurons visible in the ventral surface of the ganglion. (A) Distribution of firing frequencies of neurons visible in the ventral surface. The two peaks in the histogram indicate the existence of a population of neurons with an irregular firing rate below 0.3 Hz and of several neurons firing at a frequency above 1 Hz. These neurons were Retzius, AP, AE, 251, T, 153, Leydig cell. Data collected from 12 experiments, each of them providing optical recordings for at least 30 sec for a cumulative recording time of 8 min. The windows of the histogram varied logarithmically. (B) The cross-correlation 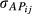 of the firing of neuron *i* and neuron *j* computed at a 100 and 1000 msec window. Values of 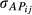 vary between 0 and 1 according to the color-coded scale shown at the right of the panel. In the window of 100 msec only the two Retzius cells have a significant positive value of 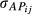. (C) Smoothed optical recordings from the pairs of Retzius, Anterior Pagoda (AP) neurons, Annulus Erector (AE) neurons, and neurons 153. Smoothing was obtained by averaging optical traces on a time window of 100 msec. Smoothing was performed to eliminate spikes and to isolate the graded and slow component of the signal. (D) The cross-correlation 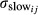 of slow signals obtained from the smoothed optical recordings in C. Values of 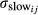 vary between −1 and 1 according to the color-coded scale shown at the right of the panel. Only the two Retzius cells have a positive value of 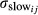. The almost oscillatory signals from the pairs of AP neurons, AE neurons, and neurons 153 are not in-phase.

We analyzed the firing pattern of neurons imaged on the ventral surface of the ganglion. We performed several dozens of experiments in which spikes could be clearly seen; in seven experiments, we detected spikes in more than 12 neurons and computed the distribution of the firing frequency of these neurons (Fig. 4A). In each one of these seven experiments we identified: a group of neurons with firing frequencies below 0.5 Hz such as neurons 216, 222, and 261, plus other neurons with firing frequencies in the range of 1–5 Hz, such as Retzius, and neurons AP, AE, 50, and 153. We rarely observed a neuron firing below 0.5 Hz for some minutes and subsequently increasing its firing frequency above 2–3 Hz. Neurons visible in the ventral surface of the ganglion either fired almost rhythmically or fired hardly at all. The regularity of these two classes of spontaneous firing was measured as two peaks in the histogram of firing rates (Fig. 4A). Occasionally, mechanosensory neurons, primarily T cells, were seen firing bursts of spikes.

We analyzed the degree of correlated firing of neurons present on the ventral surface of the ganglion, by computing the cross-correlations, 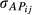, for all neuron pairs, using two different windows, 100 and 1000 msec (Fig. 4B). In the 100 msec window, only the firing of the two Retzius cells showed a large value close to 0.8, whereas all other pairs had cross-correlation values lower than 0.3. When the window was increased to 1000 msec, 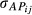 became larger than 0.6 for several pairs of neurons, including the two AEs, the two APs, and the two 153 neurons.

We obtained optical recordings of the firing of the two Retzius cells, the two APs, the two AEs, and the two 153 neurons in 6 ganglia and, in all these experiments, 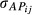 increased significantly (one-way analysis of variance (ANOVA) *P* < 0.05, followed by Tukey's post hoc test) when the window increased from 100 to 1000 msec and entries of 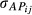 for these pairs of cells were higher than 0.6. Neurons with a positively correlated firing at a window of 1000 msec also fired at a high frequency. To determine whether the observed positive value of 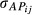 for the spikes was correlated with membrane potential changes, we analyzed the degree of correlation of their slow changes of membrane potential using equation (2). We filtered the spikes from the optical recordings by smoothing signals in a widow of 50 msec (red traces of Fig. 4C), then we computed the cross-correlation matrix between all pairs of the smoothed optical signals. Although the pair of Retzius cells had a cross-correlation value close to 0.8, in agreement with their strong electrical coupling (Hagiwara and Morita 1962) the pair of AP cells had a cross-correlation value close to 0 (Fig. 4D), indicating that their membrane potential oscillated in random phases. Also the pairs of AE and neurons 153 did not show any clear sign of correlation or anticorrelation among the graded signals recorded from their cell body.

A large fraction of leech neurons were either nonspiking neurons or had spikes restricted to the dendritic arborization and their amplitude in the soma did not exceed 2–5 mV (Ort et al. 1974; Kristan and Calabrese 1976; Brodfuehrer and Friesen 1986a,b; Brodfuehrer et al. 1995; Kristan et al. 2005). Because VF2.1.Cl can reliably detect voltage changes of 4 mV, we decided to analyze signals originating from all visible cell bodies of neurons in the ventral surface (Fig. 3A). Changes of optical signals could not only be caused by changes of membrane potentials but also by two kinds of artifacts: first sudden fluctuations in the light emitted by the illuminating lamp and second by small movements of the preparation. The first kind of artifact is present in the optical recordings from all imaged neurons and therefore it can be easily identified. The second kind of artifact produces optical signals obtained from two halves of the cell body to be in antiphase. Therefore, we considered slow changes of optical signals as reporting slow changes of voltage those that were restricted to specific neurons – not caused by lamp glitches – and those that had the same time course when obtained from nonoverlapping regions of the cell body. With these criteria, we obtained 105 optical recordings from the ventral surfaces of ganglia in 11 experiments. The value of 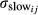 for pairs of neurons visible in the ventral surface varied between −0.3 and 0.3 and only for the two Retzius cells 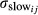 was higher than 0.7 and typically close to 0.85 (see Fig. 4D). The value of 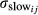 for the pair of Retzius cells was significantly higher than the value for all the other neuron pairs (one-way ANOVA *P* < 0.05, followed by Tukey's post hoc test).

The results of Figures 3and 4 show that the great majority of neurons imaged on the ventral side of the ganglion are silent at rest and do not have any significant spontaneous activity. Some other neurons fire spikes vary either rarely or almost periodically but do not have bursts of spikes, as often observed during extracellular recordings from the nerve.

### Optical recordings from the dorsal surface

**Figure 5:**
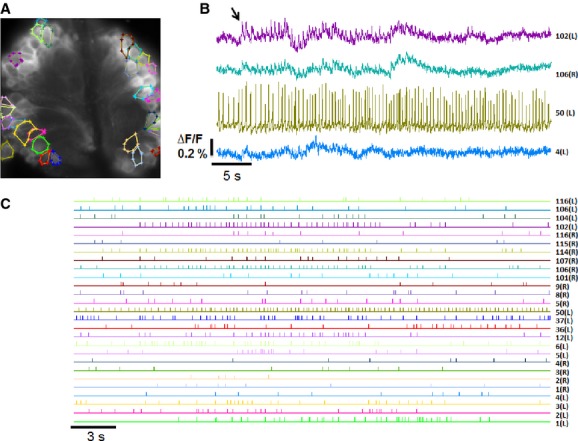
Optical recordings (ΔF/F) of the spontaneous electrical activity from the dorsal surface of a leech ganglion. (A) An image of a dorsal surface of a leech ganglion from which the optical recordings shown in B were obtained. The color of each line encircling a neuron in A corresponds to the optical recordings in B. (B) ΔF/F recordings obtained during the spontaneous activity of the ganglion. Optical recordings were processed as described in the Methods section. (C) Raster plots of detected spikes from the obtained ΔF/F recordings. Neurons were identified according to their location in the ganglion following the atlas of leech neurons (Muller et al. 1981). The letters R and L in parenthesis indicate whether the neuron was located on the right or left side of the ganglion. Neurons were identified on the basis of their location following the leech neuron atlas (Muller et al. 1981). As neurons in desheathed ganglia can be displaced from their original location, the identification of some neurons could be erroneous.

We imaged also the dorsal surface of the ganglion, where the cell bodies of most leech motoneurons are visible (Fig. 5A).

Although spikes recorded from the soma of these motoneurons do not exceed 5–7 mV (Ort et al. 1974; Kristan and Calabrese 1976; Brodfuehrer and Friesen 1986a,b; Brodfuehrer et al. 1995; Kristan et al. 2005), we could clearly detect spikes from our optical recordings (Fig. 5B). In contrast with what we observed on the ventral surface of the ganglion, motoneurons fired spikes in bursts, and a slow upward optical signal was often seen to initiate a burst of spikes (see arrow in Fig. 5B)**.** For instance, motoneurons 102, 114, and 116, after a quiet period lasting some tens of seconds, started to fire spikes, following an upward deflection of optical traces, caused by a depolarization of these motoneurons. While the burst of spikes in motoneurons lasted for 10–20 sec, neuron 50 fired spikes regularly at a rate of 2–4 Hz. From these optical recordings the raster plot of spikes (Fig. 5C), the concomitant firing of 27 motoneurons was observed. In other experiments, motoneurons 1, 2, 3, 4, 5, and 7 produced bursts of spikes. Only occasionally we observed simultaneous burst in several motoneurons, suggesting that bursts of motoneuron firing were segregated. Raster plots from the ventral (Fig. 3C) and dorsal surfaces (Fig. 5C) of a ganglion indicate some basic properties of the spontaneous firing of neurons in the ventral and dorsal surface: first, motoneurons – visible in the dorsal surface – do not fire spontaneously spikes at the same high rate as the Retzius cells and the AP neurons and AE motoneurons – visible on the ventral surface; second, diffuse and frequent bursts of spikes characterize the electrical activity of motoneurons visible on the dorsal surface. Therefore, the dynamics of the spontaneous electrical activity appears to be segregated with distinct properties in the ventral and dorsal surfaces of the ganglion.

### Correlation pattern of slow signals from motoneurons

**Figure 6:**
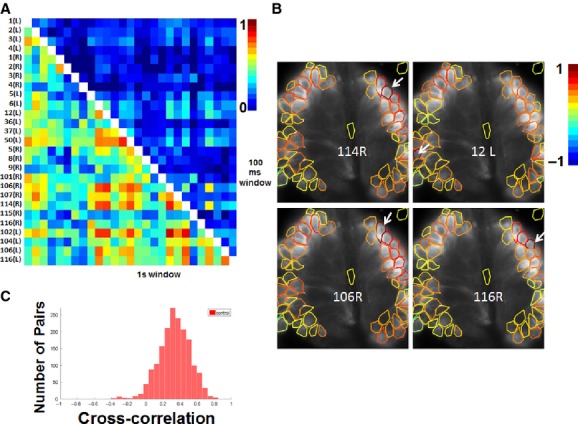
Correlation structure of electrical activity of neurons visible in the dorsal surface of the ganglion. (A) The cross-correlation 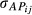 of the firing of neuron *i* and neuron *j* computed within a size window of 100 and 1000 msec. Entries of 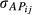 vary between 0 and 1 according to the color-coded scale shown on the right of the panel, with a window of 100 msec 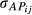 was rarely larger than 0.5 (2/351) and the window of 1 sec was occasionally larger than 0.5 (97/351). (B) Spatial profile of 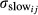 among motoneurons visible in the dorsal surface. In each panel the motoneuron indicated at the center was taken as the reference neuron *i* and 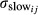 was computed for all the other visible neurons *j*. The profile of the reference neuron *i* is indicated in dark red, all the other neurons are circled with a colored line indicating the degree of cross-correlation of the graded activity measured by 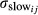 using the color-coded scale at the right of the panel. (C) distribution of values of 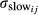 measured from neurons visible in the dorsal surface of the ganglion. Data from three experiments.

We analyzed the degree of correlated firing among pairs of neurons visible in the dorsal surface of leech ganglia (Fig. 6A). The value of 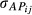 computed in a window of 100 msec was consistently low and rarely exceeded 0.5. Also within the larger window of 1000 msec, the firing was very poorly correlated and we did not find any pair of neurons showing an appreciable degree of correlated or synchronous firing. The degree of simultaneous firing was significantly lower than what observed among pairs of neurons visible on the ventral surface.

The analysis of the cross-correlation of slow optical signals shows a moderate degree of cross-correlation among neurons of the dorsal surface (Fig. 6C). Data from more than 1000 pairs of optical signals from the cell body of neurons on the dorsal surface of the ganglion indicate a mean value of the cross-correlation of 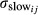 equal to 0.3 with only occasional negative entries.

Optical recordings of the spontaneous activity with fast transients identified as spikes from at least 12 different neurons were obtained in more than eight experiments from the ventral surface and nine experiments from the dorsal surface of the ganglion. In these experiments, we always detected spikes from Retzius, AE, and AP neurons, as their amplitude is relatively large. Smaller spikes originating from neurons 216, 222, and 261 were detected in five experiments when neurons were imaged from the ventral surface. In all experiments (9) imaging neurons on the dorsal surface, the fraction of entries of 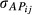 larger than 0.5 within the window of 100 and 1000 msec was smaller than 5% and 30%, respectively.

### Simultaneous electrical recordings from motoneurons and neurons in the ventral surface

The optical recordings shown in Figures 3 and 5 show that Retzius cells, AP, and AE neurons fire spikes almost periodically, while motoneurons located on the dorsal surface do not fire periodically, but alternate bursts of elevated firing with quiet periods during which no spikes are detected. These observations suggest that the spontaneous dynamics of leech neurons is segregated and that there are groups of neurons firing almost repetitively and neurons in the dorsal surface firing in bursts. In order to establish this observation it is desirable to have simultaneous recordings from neurons of the ventral and dorsal surfaces of the ganglion. For technical reasons it is not easy to obtain simultaneous optical recordings from the ventral and dorsal surfaces of a leech ganglion, but it is possible to record simultaneously the electrical activity of motoneurons with their cell body in the dorsal surface of the ganglion – with suction pipettes – and of Retzius, AE, and other neurons visible in the ventral surface with intracellular electrodes.

**Figure 7:**
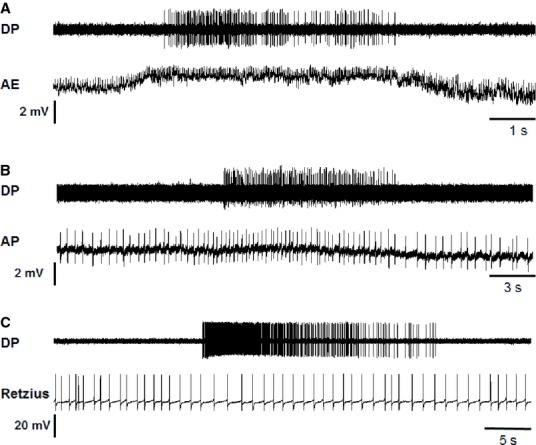
Simultaneous electrical recordings during bursts of spikes. (A) Simultaneous intracellular recording from an Annulus Erector neuron (lower trace) and extracellular recording with a suction pipette from a DP nerve (upper trace). During the burst observed in the extracellular recording the AE neuron depolarized by 2–4 mV and increased its firing rate. (B) Simultaneous intracellular recording from neuron AP (lower trace) and extracellular recording from a DP nerve (upper trace). During the burst of spikes observed in the extracellular recording the neuron AP increased its firing rate. (C) Simultaneous intracellular recording from a Retzius cell (lower trace) and extracellular recording from a DP nerve (upper trace). During the burst of spikes observed in the extracellular recording the Retzius cell did not appreciably change either its firing rate or its membrane potential.

Figure 7 illustrates simultaneous extracellular recordings from a DP nerve and intracellular recordings from neurons visible in the ventral surface of the ganglion. The AE neuron fires spikes at a high rate (around 10 Hz; Fig. 7A), similar to what is seen with optical recordings (Fig. 3). When a burst of spikes was recorded from the DP nerve, the membrane potential of the AE neuron depolarized by 2–4 mV and slightly increased its firing rate. Changes of the membrane potential of AE neurons usually preceded by 200–600 msec the onset of bursts recorded from DP nerves but not always: in some occasions (2/15 bursts) the burst in the DP nerve initiated ∼300 msec before the occurrence of a clear change of the membrane potential in AE neurons. When the AP neuron was impaled and a burst of spikes was observed on the DP nerve, its membrane potential depolarized by 1–2 mV and its firing rate increased (Fig. 7B). We impaled several Retzius cells under similar conditions and when a burst of spikes was observed in the DP nerve (Fig. 7C), its spontaneous firing rate did not significantly change. We also impaled another spontaneously firing neuron, tentatively identified as neuron 152 and when a burst of APs was observed on the DP root, its membrane potential hyperpolarized by 2–4 mV and its firing rate substantially decreased. These electrical recordings confirm the observation that some leech neurons fire spikes almost periodically and other neurons fire spikes primarily in bursts.

Changes of the resting membrane potential of impaled neurons could precede (see Fig. 7A) or follow (see Fig. 7B) the initiation of APs bursts measured on the DP nerve. This variability was observed during continuous recordings from AEs, APs, and other neurons and indicates that the initiation of bursts is not a deterministic process but it is more likely a random event that could be described by self-organized criticality (Mazzoni et al. 2007).

As shown in Figures 7, in contrast to what observed in the ventral side, neurons imaged on the dorsal side have a significant spontaneous activity and often fire spikes in bursts, in agreement with extracellular recordings from nerve terminals.

### Optical recordings in the presence of serotonin

We have also analyzed the spontaneous activity of leech neurons in the presence of 20 μmol/L of serotonin while imaging both the ventral and dorsal surfaces of the ganglion.

**Figure 8:**
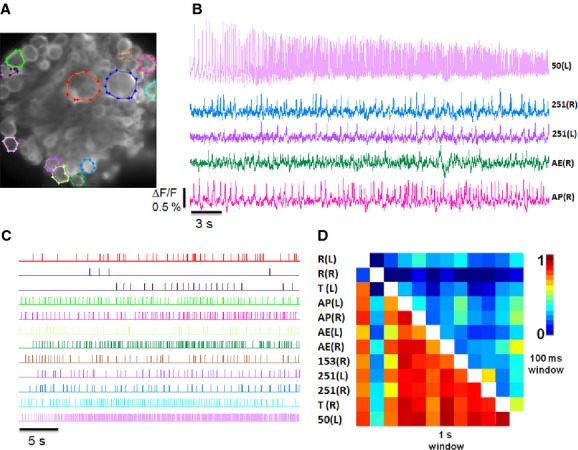
Optical recordings of the spontaneous electrical activity from the ventral surface of a leech ganglion in the presence of 20 μmol/L serotonin. (A) An image of a ventral surface of a leech ganglion from which the optical recordings shown in B were obtained. The color of each line encircling a neuron in A corresponds to the optical recordings in B. (B) ΔF/F recordings obtained during the spontaneous activity of the ganglion. (C) Raster plots of spikes detected in the ΔF/F recordings. (D) The cross-correlation 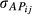 of the firing of neuron *i* and neuron *j* computed at a 100 and 1000 msec window size. Entries of 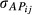 vary between 0 and 1 according to the color-coded scale shown at the right of the panel. In the presence of serotonin, Retzius cells hyperpolarize and stop firing, but the great majority of the other neurons fire more robustly and their firing appears to be more correlated at the larger window size of 1000 msec as indicated by the measured larger entries of 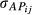. Some neurons, such as Retzius cells stop firing spikes in the presence of serotonin while other neurons, which were silent in control conditions, start firing in the presence of serotonin and therefore it is difficult to determine precisely how the matrix 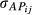 changes from control conditions and when serotonin is added.

Two minutes after adding serotonin to the extracellular medium bathing the ganglion, we started optical recordings. When the ventral surface was imaged (Fig. 8B) optically recorded spikes from the Leydig neuron (or neuron 50) progressively decreased in amplitude and decreased their interspike interval, in agreement with what has already been observed when the same neuron is impaled with an intracellular electrode and serotonin is added to the extracellular medium (Dierkes and Schlue 2005). In contrast with their activity in control conditions, Retzius cells decreased their firing rate and eventually became silent, as already observed during intracellular recordings (Nusbaum and Kristan 1986; Dierkes and Schlue 2005).

Within a window of 100 msec, entries of 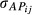 were rarely large and in fact only less than 8% of them were larger than 0.5 (Fig. 8D). However, at the window of 1000 msec, most of the entries of 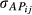 were larger than 0.5 with the exception of entries related to Retzius cells: indeed the pair of Retzius cells, in the presence of serotonin, reduce their firing rate and eventually become silent often loosing their mutual electrical coupling and entries of 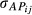 corresponding to these cells became small. In all eight experiments in which more than eight neurons were observed firing spikes in the presence of serotonin, entries of 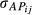 for pairs of neurons visible in the ventral surface were larger than those in control conditions (one-way ANOVA *P* < 0.05, followed by Tukey's post hoc test).

**Figure 9:**
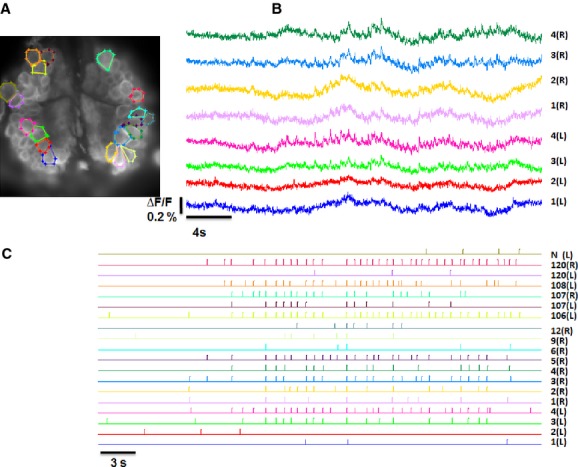
Optical recordings (ΔF/F) of the spontaneous electrical activity from the dorsal surface of a leech ganglion in the presence of 20 μmol/L serotonin. Panels A, B, and C as in Figure 5. In the presence of serotonin, many more neurons produce simultaneous bursts of spikes.

We also recorded the optical activity in the presence of serotonin from the dorsal surface (Fig. 9) where slow changes of the optical signals were more evident (Fig. 9B), indicating the occurrence of larger changes of the membrane voltage. In this case, the excitatory motoneurons 3 and 4 of both sides had a long burst of spikes (Fig. 9B) as did most of the neurons on the dorsal surface, as shown in the associated raster plot (Fig. 9C). In the presence of serotonin, spontaneous bursts of spikes in neurons visible on the dorsal surface lasted longer and activated more motoneurons than in control conditions.

**Figure 10:**
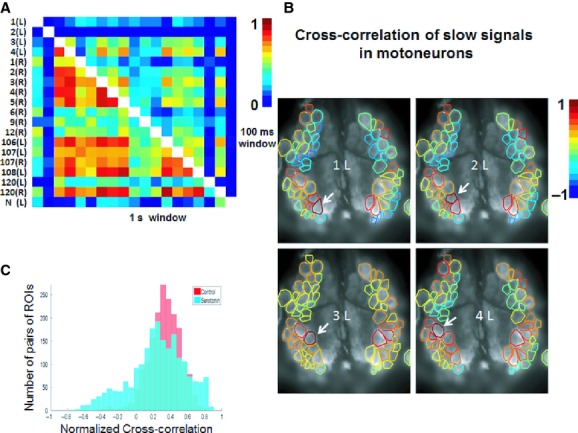
Correlation structure of electrical activity of neurons visible in the dorsal surface of the ganglion in the presence of 20 μmol/L Serotonin. Panels A, B, and C as in Figure 6. In C data collected from four experiments. Observe that in the presence of Serotonin several pairs of motoneurons have higher entries of 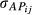 (see panel A) and several pairs of motoneurons have entries of 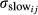 above 0.6 or below −0.2.

The analysis of the cross-correlation of slow optical signals shows a significant fraction of pairs of neurons with a negative cross-correlation (see neurons with a blue circle in Fig. 10B).

Neurons around the arrow in Figure 10B are excitatory and inhibitory motoneurons of the dorsal and ventral muscles. The excitatory motoneurons 3 and 4 are highly correlated as well as the pair of inhibitory motoneurons 1 and 2 (Kristan and Calabrese 1976; Willard 1981) but inhibitory and excitatory motoneurons are pairwise poorly correlated. Comparison of collected data of cross-correlation entries of pairs of neurons in control conditions (red histogram) and in the presence of serotonin (Blue histogram) in (Fig. 10C) indicate that serotonin induces a more structured pattern of correlation of slow optical signals, associated to correlated or anticorrelated slow changes of membrane potential in leech neurons. Optical recordings of the spontaneous activity in the presence of serotonin were obtained in more than eight experiments from the ventral surface and six experiments from the dorsal surface of the ganglion. In all these experiments Retzius cells were either silent or exhibited a significantly lower firing rate and spikes from the Leydig motoneuron decreased their amplitude during recording. In four experiments imaging the dorsal surface of the ganglion the fraction of entries of 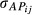 larger than 0.5 at the window of 100 and 1000 msec was larger than 5% and 30%, respectively, therefore suggesting that serotonin increased the degree of correlated firing in respect to control conditions (one-way ANOVA *P* < 0.05, followed by Tukey's post hoc test).

## Discussion

In the present manuscript, we have analyzed the simultaneous electrical activity of some dozens of neurons in the leech ganglion, by using the newly developed voltage-sensitive dye VF2.1.Cl (Miller et al. 2011). Because of its properties, this dye allows the detection of spikes with a small amplitude as those present in the cell body of most leech neurons (Ort et al. 1974; Kristan and Calabrese 1976; Brodfuehrer and Friesen 1986a,b; Brodfuehrer et al. 1995; Kristan et al. 2005). This spontaneous electrical activity is segregated in three main groups: a group of neurons firing almost periodically but not in synchrony, a group of neurons firing sparsely and randomly, and a group of neurons firing bursts with varying duration and size. These three groups interact with each other only weakly and their dynamics appear segregated. This segregation – observed in all investigated ganglia – could not be revealed by extracellular recordings from the nerves or by the simultaneous intracellular electrical recordings with 2 or 3 microelectrodes and could be detected only by this new generation of VSDs.

### Segregation of the spontaneous activity

As shown in Fig. 3 and 8, when the ventral surface of the ganglion is imaged, it is possible to visualize the cell body of as many as 80 neurons. Although we are able to detect optical signals associated to spikes with an amplitude of 4–6 mV (see Fig. 2), we detected changes of emitted fluorescence only in a limited number of visualized neurons. When the leech ganglion is involved in the generation of the electrical activity of a relevant behavior such as swimming or crawling, reliable optical signals are observed from a much larger portion of visualized neurons (Briggman et al. 2005; Briggman and Kristan 2006), suggesting that a large portion of neurons present in the ventral side do not have a significant spontaneous electrical activity. In the great majority of successful experiments we observed an almost periodic and spontaneous firing of the two Retzius cells, the two APs and AEs neurons, and of some additional neurons such as neurons 153 and 158. We occasionally observed also the spontaneous firing of mechanosensory neurons such as T, P, and N cells. When the cell body of other neurons in the ventral surface was imaged, we rarely detected optical signals indicating the occurrence of a spike. As shown in Figure 2B, we were able to detect optically from the cell body of imaged neurons spikes with amplitude of 4 mV. Therefore, the spontaneous electrical activity of the great majority of neurons visible in the ventral surface of leech ganglia is either very limited and almost absent or is segregated into their dendritic branching. A different picture emerges when neurons in the dorsal surface are imaged (Fig. 5): optical signals associated to the occurrence of spikes could be recorded – in successful experiments – from the great majority of imaged neurons. These neurons do not usually fire in a repetitive or periodic mode in bursts of 3–10 spikes lasting for some seconds, as confirmed by extracellular recordings with suction pipettes from the nerves shown in Figure 7. As shown in Figures 3 and 7, the spontaneous firing of the Retzius, APs, AEs is almost periodic and does not occur in bursts, as in motoneurons visible in the dorsal surface. Therefore, under control conditions, the spontaneous activity of neurons in a leech ganglion has three different regimes: (1) a repetitive and almost periodic firing around 1 Hz; (2) a sparse and random firing, and (3) a firing in bursts of varying duration and size. These different regimes involve specific neurons and a given neuron does not change its firing regime.

Simultaneous electrical recordings from the nerves and from Retzius, AP, and AE neurons (Fig. 7) show that when a burst occurs in motoneurons, this burst has a limited effect on the electrical activity of these neurons. As shown in Figure 7, changes of the resting membrane potential of the impaled neurons visible on the ventral surface (AEs, APs, Retzius cells,….) could precede or follow the initiation of bursts, in agreement with the notion that bursts dynamics is not deterministic but reminiscent of self-organized criticality (Mazzoni et al. 2007). Therefore, the spontaneous electrical activity of leech neurons appears to be segregated in three distinct groups with their own regime.

### Subthreshold interactions among leech neurons

The dye VF2.1.Cl is not only able to signal the occurrence of a spike with amplitude larger than 4 mV, but – as shown in Figure 2 – can detect changes of the membrane potential larger than ∼4–6 mV. Therefore, imaging with this dye offers the possibility to evaluate and identify the extent of subthreshold interactions among leech neurons. The analysis of 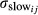 among pairs of neurons visible in the ventral surface shows a strong positive correlation among the pair of Retzius cells and only some rather weak and possibly negligible subthreshold couplings among other pairs of neurons. In contrast, neurons visible in the dorsal surface, exhibit some degree of subthreshold positive correlation and graded optical signals recorded from neighboring motoneurons have often a positive value of 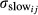. In the presence of serotonin, neurons become more coupled, with the exception of Retzius cells (see Fig. 8). The comparison of 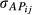 of neurons visible in the ventral surface indicates an increased correlated firing in the presence of serotonin (Fig. 8D) with respect to what is observed in control conditions (Fig. 4B). Neurons visible in the dorsal surface of the ganglion, which usually have positive entries of 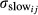 at around 0.3 become either more strongly positively coupled or exhibit negative entries of 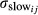. This effect is consistent with the swimming behavior induced in a chain of ganglia by micromolar amounts of serotonin (Kristan et al. 2005).

### The effect of serotonin

Our optical recordings of the perfusion of serotonin (20 μmol/L) of a single isolated leech confirms several previous observations (Nusbaum and Kristan 1986; Dierkes and Schlue 2005): indeed, the amplitude of Retzius cells spikes decreases and eventually these cells become silent and their electrical coupling is weakened (Fig. 8). Slow signals from neurons visible in the dorsal surface, which in control conditions did not have large positive or negative values (Fig. 6B and C), exhibited large positive and negative values, as shown in Figure 10 B and C. It is well known that serotonin switches behaviors – such as swimming – on and off (Kristan and Calabrese 1976; Willard 1981). Therefore, the addition of serotonin to a single isolated leech ganglion creates groups of neurons with in-phase and antiphase slow changes of their membrane voltage, but the emergence of the swimming pattern – in which motoneurons fire with specific phase lags – requires the presence of a chain of ganglia.

### Possible functional role of segregation of the spontaneous activity

The segregation of the spontaneous electrical activity here described can have an important functional role in information processing in the leech nervous system. Indeed, neurons on the ventral surface are sensory neurons and interneurons, while neurons on the dorsal surface are primarily motoneurons. A highly irregular firing of spikes and/or the presence of large bursts are certainly not beneficial to the processing of sensory information. From this point of view it is not surprising that neurons devoted to the analysis, processing and filtering of sensory and particularly mechanical inputs do not have a significant spontaneous activity: indeed, it is advantageous that interneurons involved in the first stage of sensory processing do not fire spontaneously and are relatively noise free. Neurons on the dorsal surface are not sensory neurons and are primarily effectors, that is, are motoneurons.

Our results give a contribution to the current discussion on the role and properties of “stimulus driven” and “network driven” neuronal mechanisms (Benucci et al. 2009; Churchland et al. 2012). Neurons in the ventral surface of the ganglion seem to be primarily driven by sensory stimuli, while the electrical activity of neurons, and in particular motoneurons, in the dorsal surface is not only driven by sensory inputs but it is also influenced by network properties determining the occurrence of bursts. The observation of substantially higher entries of 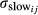 in pairs of neurons visible in the dorsal surface than in the ventral surface supports the notion that neurons in the dorsal surface are more network driven than those in the ventral surface.
